# Participation of *S.* Typhimurium *cysJIH* Operon in the H_2_S-mediated Ciprofloxacin Resistance in Presence of Sulfate as Sulfur Source

**DOI:** 10.3390/antibiotics4030321

**Published:** 2015-07-31

**Authors:** Ricardo Álvarez, Jorge Frávega, Paula I. Rodas, Juan A. Fuentes, Daniel Paredes-Sabja, Iván L. Calderón, Fernando Gil

**Affiliations:** 1 Laboratorio de Genética y Patogénesis Bacteriana, Departamento de Ciencias Biológicas, Facultad de Ciencias Biológicas, Universidad Andres Bello, República 217, Santiago 8370146, Chile; E-Mails: rh.alvareze@gmail.com (R.A.); fravegaj@gmail.com (J.F.); lcalderon@unab.cl (I.L.C.); 2 Center for Integrative Medicine and Innovative Science, Facultad de Medicina, Universidad Andres Bello, Echaurren 183, Santiago 8370071, Chile; E-Mail: paula.rodas@unab.cl; 3 Laboratorio de Microbiología, Departamento de Ciencias Biológicas, Facultad de Ciencias Biológicas, Universidad Andres Bello, República 217, Santiago 8370146, Chile; E-Mail: jfuentes@unab.cl; 4 Gut Microbiota and Clostridia Research Group, Departamento de Ciencias Biológicas, Facultad de Ciencias Biológicas, Universidad Andres Bello, República 217, Santiago 837014, Chile; E-Mail: daniel.paredes.sabja@gmail.com

**Keywords:** *cysJIH*, ciprofloxacin resistance, H_2_S production, sulfate, sulfur source

## Abstract

H_2_S production has been proposed as a mechanism to explain bacterial resistance to antibiotics. In this work, we present evidence for the role of the *cysJIH* operon in resistance to ciprofloxacin mediated by H_2_S production with different sulfate as the only sulfur source. We found that the products of the *cysJIH* operon are involved in ciprofloxacin resistance by increasing both, the levels of H_2_S and reduced thiols apparently counteracting antimicrobial-induced reactive oxygen species (ROS). This protective effect was observed only when bacteria were cultured in the presence of sulfate, but not with cysteine, as the sole sulfur source.

## 1. Introduction

In prokaryotes, sulfur can be assimilated into sulfur-containing amino acids through enzymatic fixation from inorganic sources such as sulfate [1], or from organic sources such as cysteine [2,3,4]. Since H_2_S is considered a gasotransmitter that protects neurons and cardiac muscle from oxidative stress [5,6,7], it has been hypothesized that bacterial H_2_S likewise acts as a cellular protector. In this sense, bacteria with mutations that suppress H_2_S production are sensitive to several antimicrobial compounds that exert their bactericidal activity via oxidative stress, like β-lactam antibiotics [8,9,10].

The genes of the *cysJIH* operon encode enzymes that participate in the last step of H_2_S synthesis in the sulfate assimilation pathway [11]. In *Salmonella enterica* serovar Typhimurium (*S.* Typhimurium), the role of the *cysJIH* operon in the protection against reactive oxygen species (ROS) induced by antibiotics or other compounds was demonstrated in our last work, where the sulfur source was determinant in the protection against two ROS-generating compounds (ceftriaxone and menadione) [12]. In the present study, we examined the role of *cysJIH* in the resistance to the antimicrobial activity of ciprofloxacin (CIP), a quinolone. We found that the products of the *cysJIH* operon are involved in CIP-resistance by increasing both the levels of H_2_S and reduced thiols, apparently counteracting the ROS induced by this kind of antimicrobial agents. This protective effect was observed only when bacteria were cultured in sulfate, but not with cysteine, as the sole sulfur source.

## 2. Materials and Methods

### 2.1. Bacterial Strains and Growth Conditions

*S.* Typhimurium ATCC 14028s, and Δ*cysJIH* and WT/pBAD*cysJIH* derivatives were described previously in Álvarez *et* *al*. [12]. Very briefly, Δ*cysJIH* strain was obtained using cysJIHwannerF (5'-TTACTGGAACATAACGACGCATGACGACACCGGCTCCACTTGTAGGCTGGAGCTGCTTCG-3') and cysJIHwannerR (5'-ATCATACCGCGTAAGGACAATTACCCTTCATGCAGCCCGCCATATGAATATCCTCCTTAG-3') to perform the Red-Swap technique [13]. The pBAD*cysJIH* plasmid, containing the *S.* Typhimurium *cysJIH* operon under the P_ara_ promoter, was obtained as previously described [11] by cloning the *cysJIH* operon obtained by PCR using pBADcysJIHF (5'-ATGACGACACCGGCTCCACTGACTG-3') and pBADcysJIHR (5'-CCCTTCATGCAGCCCGCACTCGCGC-3') primers.

The strains were grown routinely at 37 °C in Luria Bertani broth (LB) with shaking to OD_600_ 0.45, washed 3 times with sterile PBS, and changed when required to glucose 0.4% minimal medium 9 (M9) supplemented either with sulfate (MgSO_4_ 2 mM) or cysteine (0.5 mM) as the sulfur source. When necessary, media were supplemented with sub-lethal concentrations of CIP (0.91 μM) according to MIC determination for strains in all culture media used in this work.

### 2.2. Determination of the Minimal Inhibitory Concentration (MIC) of CIP

*S.* Typhimurium ATCC 14028s, Δ*cysJIH* and WT/pBAD*cysJIH*, were grown routinely at 37 °C in Luria Bertani broth (LB) with shaking to OD_600_ 0.45, washed 3 times with sterile PBS, and diluted to OD_600_ 0.05 in LB or 0.4% glucose M9 supplemented with either sulfate or cysteine as the sulfur source. Then, 290 μl of bacteria were inoculated to a microplate containing serial dilutions of CIP. Microplates were incubated for 18 h at 37 ºC and OD_600_ were determined. MIC was considered at dilution in which every strain grown < 50% with respect to control (no toxic compound added).

### 2.3. Determination of Intracellular ROS Levels

Bacterial cultures were grown as specified above, using either sulfate or cysteine as the sole sulfur source. When necessary, bacterial cultures were exposed to CIP for 20 min. Protocol was performed according to Alvarez *et* *al*. [12].

### 2.4. Determination of Superoxide Dismutase (SOD) Activity

Bacterial cultures were grown as specified above. When necessary, bacterial cultures were exposed to CIP for 20 min. SOD activity was assessed by measuring the inhibition of the photochemical reduction of nitro blue tretrazolium (NBT) from crude extracts as previously described [14].

### 2.5. Determination of Reduced Thiols

Bacterial cultures were grown as specified above. When necessary, bacterial cultures were exposed to CIP for 20 min. Reduced thiols were quantified using Ellman’s reagent (DTNB) according to protocol described in Alvarez *et* *al*. [12].

### 2.6. H_2_S Production

To monitor H_2_S production in *S.* Typhimurium WT and mutant strains, we used the lead acetate detection method [9]. When necessary, bacterial cultures were exposed to CIP for 20 min. Stained paper strips were quantified with ImageJ software. The results were normalized per OD.

### 2.7. Statistics

*p* Values were calculated according the ANOVA test using Bonferroni post-hoc. Values *p* < 0.05 were considered statistically significant.

## 3. Results and Discussion

### 3.1. Deletion of S. Typhimurium cysJIH Results in Increased Intracellular Levels of ROS and Decreased SOD Activity in Presence of Ciprofloxacin When Bacteria Were Cultured with Sulfate as the Sole Sulfur Source

**Figure 1:**
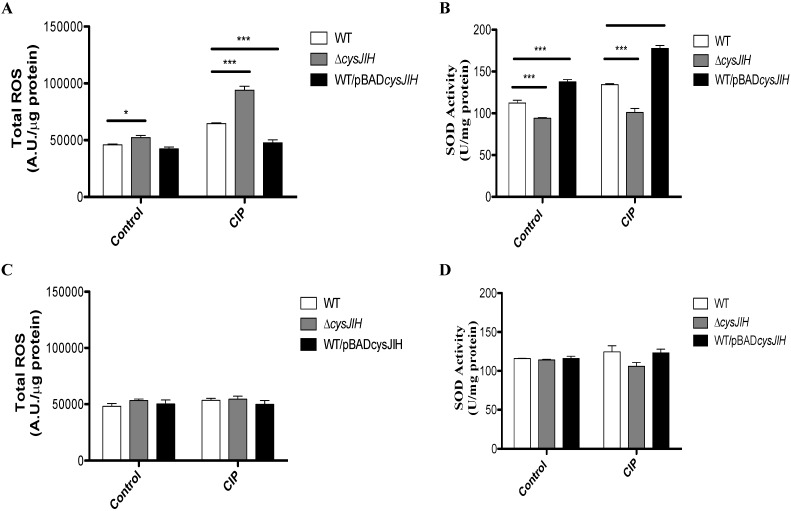
Total reactive oxygen species (ROS) and superoxide dismutase (SOD) activity in *S*. Typhimurium WT and mutant derivative. *S.* Typhimurium strains were grown in LB medium to OD_600_ 0.45 and subsequently cultured in M9 + sulfate (**A**,**B**) or M9 + cysteine (**C**,**D**), treated or not with CIP. (**A**,**C**) total ROS; (**B**,**D**) SOD activity. For all graphics, white bars: *S.* Typhimurium WT; grey bars: *S.* Typhimurium Δ*cysJIH*; black bars: *S.* Typhimurium WT/pBAD*cysJIH*. Experiments were repeated six times and asterisks represent statistically significant differences as compared with *S.* Typhimurium WT in each treatment (* *p* < 0.05; ** *p* < 0.01; *** *p* < 0.001).

To evaluate the role of *cysJIH* in the resistance to ciprofloxacin (CIP), we determined the minimal inhibitory concentration (MIC) of *S*. Typhimurium WT and Δc*ysJIH* in sulfate and cysteine minimal media. As shown in Table 1, *S.* Typhimurium Δ*cysJIH* strain was more sensitive to CIP compared with the WT strain in sulfate medium. This phenotype was reverted in the *S.* Typhimurium Δ*cysJIH* complemented with wild-type *cysJIH*. Furthermore, the *S.* Typhimurium WT/pBAD*cysJIH*, a *cysJIH*-overexpressing strain, exhibited even more resistance to CIP than the WT. In cysteine medium, no changes were observed for any strain. Previously, we reported that *cysJIH* contributes to diminish ROS levels induced by the exposure to antimicrobial agents such as menadione and ceftriaxone by increasing the SOD activity, the levels of reduced thiols, and H_2_S. This effect was only observed when bacteria were cultured with sulfate as the sole source of sulfur [12], strongly suggesting that *cysJIH* also contribute to CIP resistance by diminishing ROS. To determine the role of *cysJIH* in the CIP resistance, we measured oxidative stress markers in *S.* Typhimurium WT, *S.* Typhimurium Δ*cysJIH*, and *S.* Typhimurium WT/pBAD*cysJIH* in the presence of this antibiotic. As shown in Figure 1, exposure to CIP increased total ROS (Figure 1A) and decreased SOD activity (Figure 1B) in a Δ*cysJIH* background only when bacteria were cultured with sulfate as the sole sulfur source. In contrast, when bacteria were cultured with cysteine as the sole sulfur source, exposure to CIP had no effect on these same parameters (Figure 1C,D). This result supports the contribution of ROS in the CIP antimicrobial activity (compare Figure 1 and Table 1). Accordingly, we found similar results with ceftriaxone and menadione [12]. Kohanski *et* *al*. [15] proposed that some bactericidal antibiotic increases the intracellular levels of ROS. In this sense, several bacterial species could be able to use H_2_S as a cellular protector to increase resistance to ROS triggered by bactericidal antibiotics [9]. Our results confirm that in a Δ*cysJIH* strain, ROS response after exposure to CIP is diminished probably due to lower SOD activity in media with sulfate as the sole sulfur source. This effect, as suggested by Shatalin *et* *al*. [9], is probably due to a deficient H_2_S production and hence a less protector effect. The role of H_2_S in protection to ROS producing agents has been previously suggested. For instance, *S.* Typhimurium Δ*cysK* mutant, which could accumulate H_2_S, is 3-fold more resistant to ciprofloxacin than the WT strain [16,17]. Moreover, upon increased cysteine concentrations, H_2_S can act as a reducing agent that fuels the Fenton reaction [18]; consequently a transient depletion of free cysteine to produce H_2_S could allow bacteria to resist the oxidative stress. Altogether, the results presented associates *cysJIH* with ROS and ciprofloxacin resistance, as described in our previous work with ceftriaxone [12].

**Table 1:** Minimal Inhibitory Concentration (MIC) determination of strains used in this study

Strain *	MIC CIP (μM) sulfate	MIC CIP (μM) cysteine
*Salmonella* Typhimurium ATCC 14028s	3.64	3.64
*Salmonella* Typhimurium ATCC 14028s Δ*cysJIH*	1.82	3.64
*Salmonella* Typhimurium ATCC 14028s Δ*cysJIH*/pBAD*cysJIH*	3.64	3.64
*Salmonella* Typhimurium ATCC 14028s WT/pBAD*cysJIH*	5.46	3.64

* All bacteria were treated with ciprofloxacin (CIP) in both M9-sulfate and M9-cysteine media; all determinations were performed 6 times.

### 3.2. CIP Induces the Accumulation of Reduced Thiols and H_2_S in a cysJIH-Dependent Manner with Sulfate as the Sole Sulfur Source

**Figure 2:**
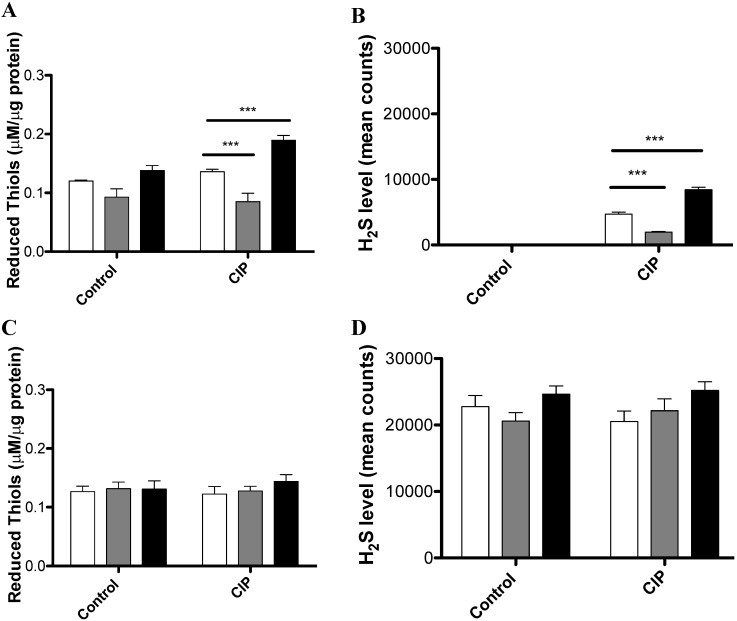
Reduced Thiols and H_2_S production in *S*. Typhimurium WT and mutant derivative. *S.* Typhimurium WT and Δ*cysJIH* strains were grown in LB medium to OD_600_ 0.45 and subsequently cultured in M9 + sulfate (**A**–**C**) or M9 + cysteine (**C**,**D**), treated or not with CIP for 20 min. (**A**,**C**) total thiols; (**B**,**D**) H_2_S levels. For all graphics: White bars: *S.* Typhimurium WT; grey bars: *S.* Typhimurium Δ*cysJIH*; black bars: *S.* Typhimurium WT/pBAD*cysJIH*. Data were normalized to the control in which no treatment was used. Experiments were repeated six times and asterisks represent statistically significant differences as compared with *S.* Typhimurium WT in each treatment (* *p* < 0.05; ** *p* < 0.01; *** *p* < 0.001).

*S*. Typhimurium Δ*cysJIH* mutant accumulated more ROS and presented decreased levels of SOD activity as compared with the *S.* Typhimurium WT, after exposure to CIP. Conceivably, this effect might be explained by a differential H_2_S accumulation in these strains as reported in our previous work [12]. To test this hypothesis, we evaluated the total reduced thiols and H_2_S levels induced by CIP in *S.* Typhimurium WT, *S.* Typhimurium Δ*cysJIH*, or *S.* Typhimurium WT/pBAD*cysJIH*. As shown in Figure 2, *S.* Typhimurium Δ*cysJIH* accumulated less reduced thiols (Figure 2A) and less H_2_S (Figure 2B, data were normalized to the control in which no treatment was amended) compared with *S.* Typhimurium WT when bacteria were cultured with sulfate in the presence of CIP. Conversely, *S.* Typhimurium WT/pBAD*cysJIH*, a strain that overexpresses *cysJIH*, exhibited a higher accumulation of reduced thiols and H_2_S (Figure 2A,B). In the case of bacteria grown in cysteine, we found that reduced thiols and H_2_S were accumulated under all of tested culture conditions, where the addition of CIP exerted no effect (Figure 2C,D). Thus, reduced thiols and H_2_S accumulation perfectly correlate with decreased ROS and increased SOD activity, as shown in our previous work [12].

Altogether, our results show that the exposure to the antimicrobial agent CIP induces H_2_S accumulation in a *cysJIH*-dependent manner when bacteria were grown with sulfate as the sole sulfur source. This provides evidence that argues in favor of a mechanism(s) of antibiotic-induced oxidative stress resistance that involves genes that participate in H_2_S production. Several experiments are required for elucidate the importance of this operon-mechanism relative to CIP resistant enzymes (DNA gyrase and topoisomerase IV) [19] or genes controlling efflux/accumulation of quinolones [20,21], all of which are prevalent and account for clinical failures to CIP and correlate with MIC break points. Such experiments would seemingly define the importance of *cysJIH* mutations to enhance or decrease CIP susceptibility. A proteomic study could be a good approach to further understand this mechanism at the molecular level, in order to control resistant strains and to develop new therapeutic strategies [21].

## 4. Conclusions

- *cysJIH* operon are involved in CIP-resistance by increasing both the levels of H_2_S and reduced thiols
- The protective effect of *cysJIH* operon was observed only when bacteria were cultured in sulfate as the sole sulfur source.
